# Supplementary material for: Precision fMRI reveals densely interdigitated network patches with conserved motifs in the lateral prefrontal cortex
Source: Neuron. Author manuscript; Available in PMC 2026 Jun 23. (PMC13289807; doi:10.1016/j.neuron.2026.04.011)
Supplement: MMC1 [file NIHMS2178885-supplement-MMC1.pdf]

Neuron, Volume *114*

Supplemental information

Precision fMRI reveals densely interdigitated network patches with conserved motifs in the lateral prefrontal cortex

Zach Ladwig, Kian Z. Kermani, Youngeun Park, Elena Housteau, Ally Dworetsky, Nathan Labora, Joanna J. Hernandez, Megan Dorn, Derek M. Smith, Derek Evan Nee, Steven E. Petersen, Rodrigo M. Braga, and Caterina Gratton

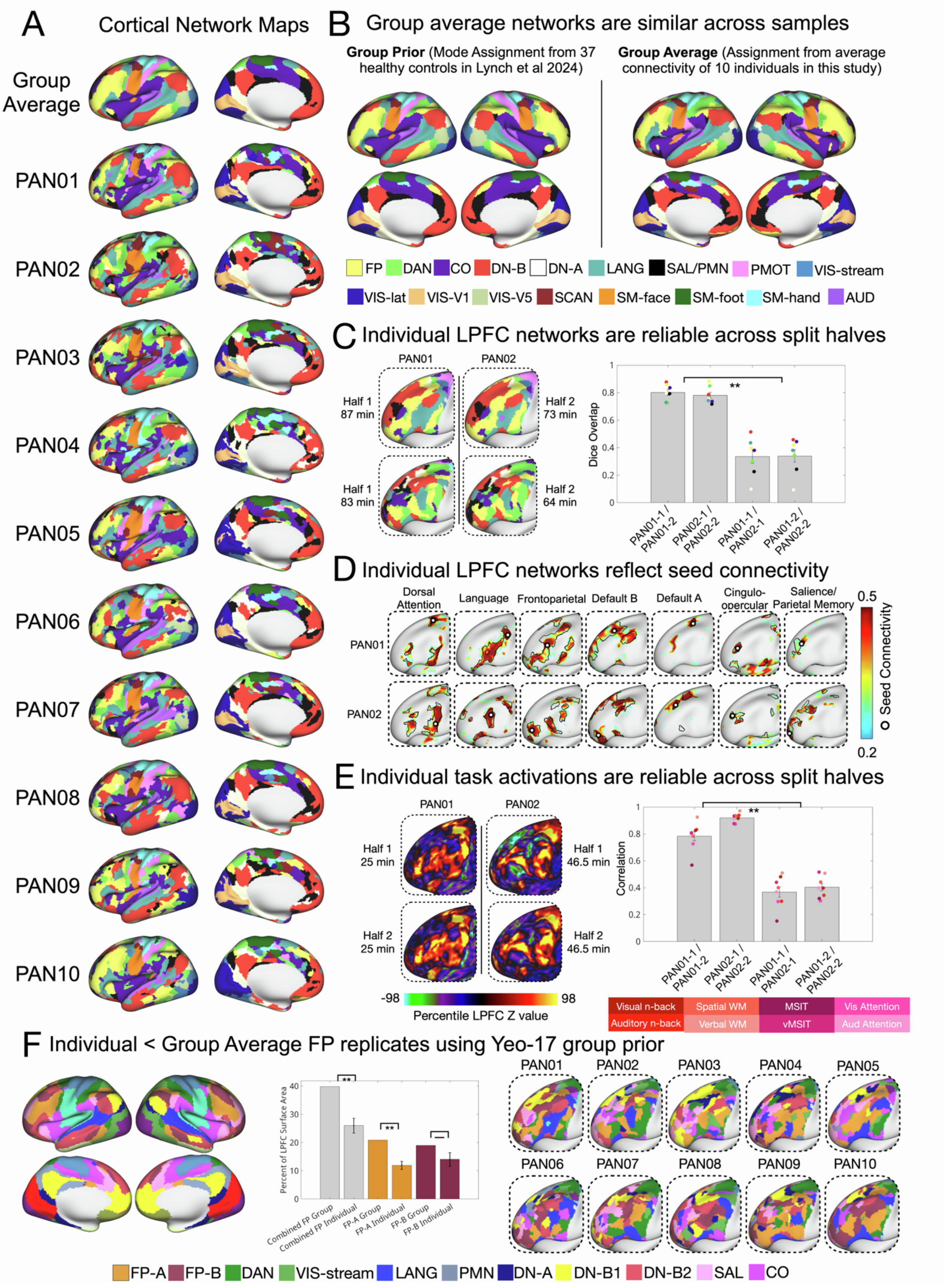

A Cortical Network Maps
Group Average
PAN01
PAN02
PAN03
PAN04
PAN05
PAN06
PAN07
PAN08
PAN09
PAN10
B Group average networks are similar across samples
Group Prior (Mode Assignment from 37 healthy controls in Lynch et al 2024)
Group Average (Assignment from average connectivity of 10 individuals in this study)
FP DAN CO DN-B DN-A LANG SAL/PMN PMOT VIS-stream
VIS-lat VIS-V1 VIS-V5 SCAN SM-face SM-foot SM-hand AUD
C Individual LPFC networks are reliable across split halves
PAN01
PAN02
Half 1 87 min
Half 2 73 min
Half 1 83 min
Half 2 64 min
Dice Overlap
PAN01-1 / PAN01-2
PAN02-1 / PAN02-2
PAN01-1 / PAN02-1
PAN01-2 / PAN02-2
D Individual LPFC networks reflect seed connectivity
Dorsal Attention
Language
Frontoparietal
Default B
Default A
Cingulo-opercular
Salience/ Parietal Memory
PAN01
PAN02
0.5
Seed Connectivity
0.2
E Individual task activations are reliable across split halves
PAN01
PAN02
Half 1 25 min
Half 1 46.5 min
Half 2 25 min
Half 2 46.5 min
Correlation
-98
98
Percentile LPFC Z value
Visual n-back
Spatial WM
MSIT
Vis Attention
Auditory n-back
Verbal WM
vMSIT
Aud Attention
F Individual < Group Average FP replicates using Yeo-17 group prior
Percent of LPFC Surface Area
Combined FP Group
Combined FP Individual
FP-A Group
FP-A Individual
FP-B Group
FP-B Individual
FP-A FP-B DAN VIS-stream LANG PMN DN-A DN-B1 DN-B2 SAL CO

Supplemental Figure S1. Individual-specific network maps and task activations are reliable and robust. Related to Figure 1.

(A) Network parcellations were generated for all ten individuals. While the main text focuses on the lateral prefrontal cortex (LPFC), parcellations were defined across the entire cortical surface. (B) A group average parcellation was generated using the average functional connectivity matrix from the ten individuals in the present study. This parcellation was highly similar to the mode parcellation derived from 37 healthy adults reported in Lynch et al. 2024[1], suggesting the group average is stable across independent samples. (C) For two high-data individuals (PAN01 and PAN02), parcellations were generated from two independent split-halves based on scanning day (odd vs. even sessions). LPFC network topographies were highly reliable across halves within individuals (mean within-individual Dice coefficient = 0.79 ± 0.05, mean between-individual Dice coefficient = 0.34 ± 0.13, $t(6) = 9.8$, $p < 0.0001$). (D) For two high-data individuals (PAN01 and PAN02), task maps were derived from two independent split-halves based on scanning day (odd vs. even sessions). Task maps were reliable across split halves within an individual (mean within-individual correlation = 0.85 ± 0.06, mean between-individual correlation = 0.39 ± 0.09, paired-samples t-test $t(7) = 21.6$, $p < 0.000001$). Visual n-back maps are shown as an example. (E) For PAN01 and PAN02, seeds were manually placed within the boundaries of each association network in the LPFC. The resulting connectivity maps closely matched the parcellated network territories. (F) Individual-specific LPFC parcellations were re-derived using an alternative group prior that included two distinct frontoparietal networks (Yeo-17 networks, FP-A and FP-B[2]). The primary finding of inflated frontoparietal representation in group-level maps was replicated (one-sample t-test: $t(9) = 5.3$, $p = 0.0005$). Data are presented as mean ± SEM. Significant ($p < 0.05$) differences are indicated with asterisks.

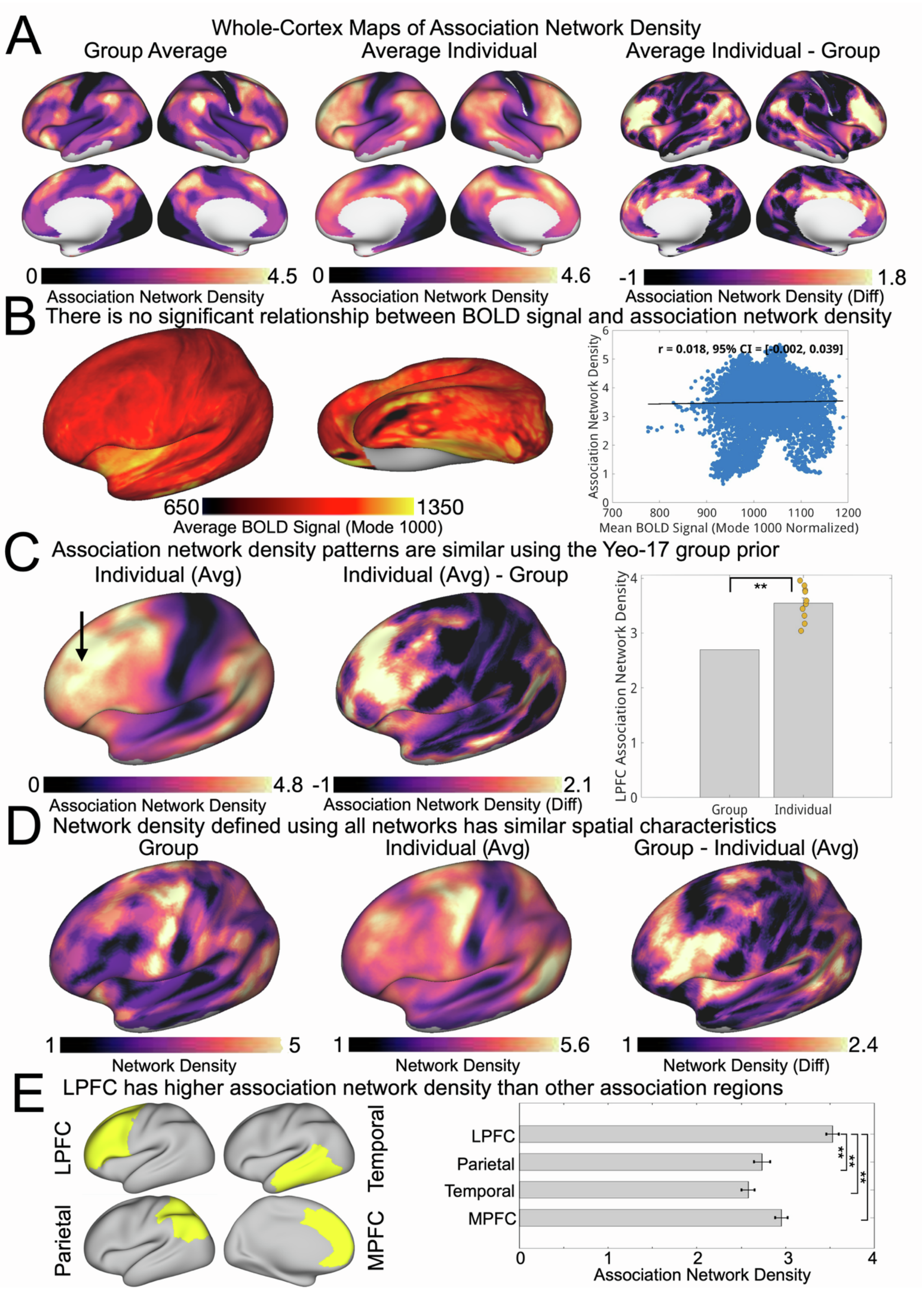
A
Whole-Cortex Maps of Association Network Density
Group Average
Average Individual
Average Individual - Group
0
4.5
Association Network Density
0
4.6
Association Network Density
-1
1.8
Association Network Density (Diff)
B
There is no significant relationship between BOLD signal and association network density
650
1350
Average BOLD Signal (Mode 1000)
r = 0.018, 95% CI = [-0.002, 0.039]
Association Network Density
Mean BOLD Signal (Mode 1000 Normalized)
C
Association network density patterns are similar using the Yeo-17 group prior
Individual (Avg)
Individual (Avg) - Group
0
4.8
Association Network Density
-1
2.1
Association Network Density (Diff)
LPFC Association Network Density
**
Group
Individual
D
Network density defined using all networks has similar spatial characteristics
Group
Individual (Avg)
Group - Individual (Avg)
1
5
Network Density
1
5.6
Network Density
1
2.4
Network Density (Diff)
E
LPFC has higher association network density than other association regions
LPFC
Temporal
Parietal
MPFC
LPFC
Parietal
Temporal
MPFC
**
**
**
Association Network Density

Supplemental Figure S2. Association network density patterns are not related to BOLD signal patterns and replicate across analytical choices. The LPFC is more network dense than other association regions. Related to Figure 2.

(A) Association network density is shown for the group-average map (left), the across-individual average (middle), and the difference between the two (right). Group averaging underestimates association network density in the rostral LPFC. (B) A measure of BOLD signal (after mode 1000 normalization) across individuals is shown across the left hemisphere cortical surface. There is no meaningful relationship between BOLD signal and association network density ($r = 0.018$, 95% CI: [–0.002, 0.039]). (C) Association network density was calculated using the Yeo-17 group prior and the finding that individual density > group density was replicated (one-sample t-test: $t(9) = 8.82$, $p = 0.00001$). (D) Network density maps were recreated counting all functional networks rather than only association networks. The group and average individual maps resemble association network density maps but also highlight regions where association networks sit close to local networks (e.g. caudal LPFC). The difference map closely resembles the association network density map. (E) ROIs were defined for three other association regions of the brain (lateral temporal, lateral parietal, and medial prefrontal cortex). The lateral prefrontal cortex exhibited higher association network density than the lateral parietal cortex (paired t-test: $t(9) = 6.0$, $p < 0.001$), the lateral temporal cortex (paired t-test: $t(9) = 10.0$, $p < 0.001$), and the medial prefrontal cortex (paired t-test: $t(9) = 8.1$, $p < 0.001$). Data are presented as mean ± SEM.

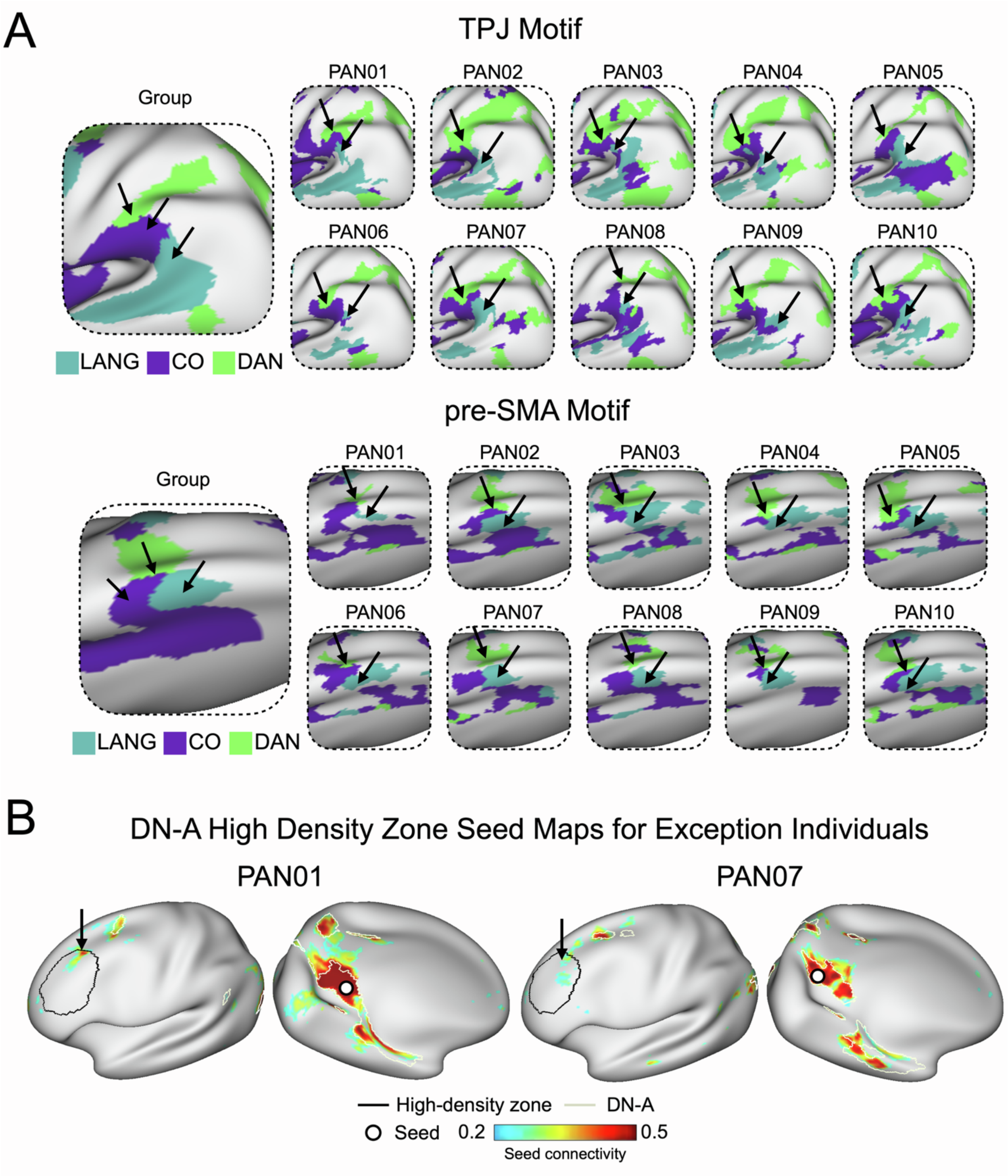
A
TPJ Motif
Group
PAN01
PAN02
PAN03
PAN04
PAN05
PAN06
PAN07
PAN08
PAN09
PAN10
LANG
CO
DAN
pre-SMA Motif
Group
PAN01
PAN02
PAN03
PAN04
PAN05
PAN06
PAN07
PAN08
PAN09
PAN10
LANG
CO
DAN
B
DN-A High Density Zone Seed Maps for Exception Individuals
PAN01
PAN07
High-density zone
DN-A
Seed
0.2
0.5
Seed connectivity

Supplemental Figure S3. Examples of network motifs and seed maps for exception individuals. Related to Figures 3 and 4.

(A) Network parcellations for each individual were manually examined for evidence of a repeating three-network motif involving the dorsal attention network (DAN), cingulo-opercular network (CO), and language network (LANG). This motif was observed in all ten individuals in both the temporoparietal junction (TPJ) and the pre-supplementary motor area (pre-SMA). In both regions, the spatial arrangement of the motif was sufficiently consistent across individuals that it is also evident in the group-average map. (B) PAN01 and PAN07 did not exhibit DN-A network territory within the rostral LPFC high-density zone based on their individual parcellations. However, manually selected seeds within DN-A in these individuals showed functional connectivity to this location. This suggests the possible presence of DN-A–related signal in this region that was not captured by the discrete parcellation boundaries. The high-density zone shown is a 15mm radius around the rostral CO region.

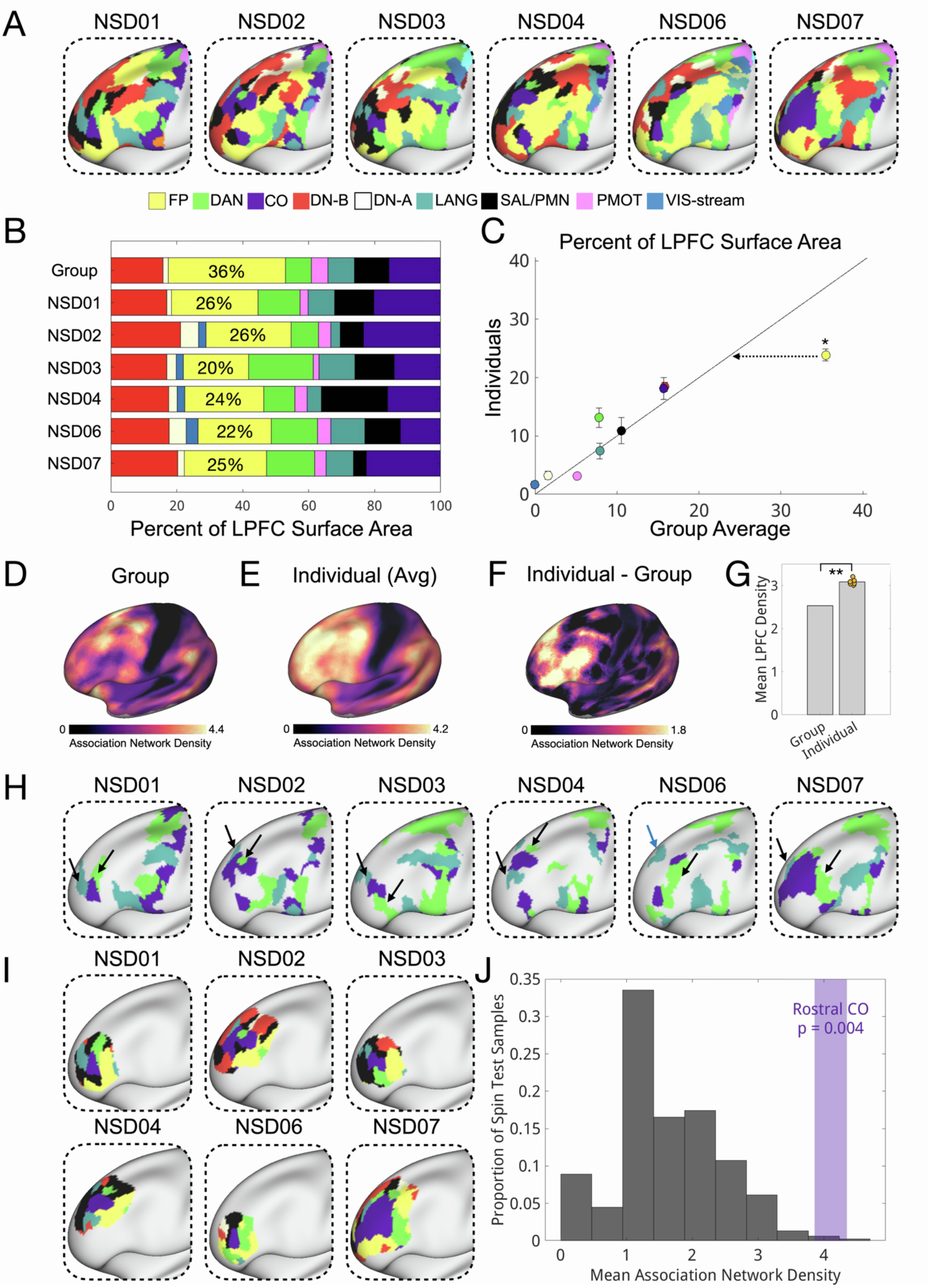

A
NSD01
NSD02
NSD03
NSD04
NSD06
NSD07
FP
DAN
CO
DN-B
DN-A
LANG
SAL/PMN
PMOT
VIS-stream
B
Group
NSD01
NSD02
NSD03
NSD04
NSD06
NSD07
36%
26%
26%
20%
24%
22%
25%
0
20
40
60
80
100
Percent of LPFC Surface Area
C
Percent of LPFC Surface Area
Individuals
Group Average
0
10
20
30
40
*
D
Group
E
Individual (Avg)
F
Individual - Group
0
4.4
4.2
1.8
Association Network Density
G
Mean LPFC Density
**
Group
Individual
H
NSD01
NSD02
NSD03
NSD04
NSD06
NSD07
I
NSD01
NSD02
NSD03
NSD04
NSD06
NSD07
J
Proportion of Spin Test Samples
Rostral CO
p = 0.004
Mean Association Network Density
0.35
0.3
0.25
0.2
0.15
0.1
0.05
0
1
2
3
4

Supplemental Figure S4. LPFC network organization results replicate in an independent publicly available precision fMRI dataset. Related to Figures 1-4.

(A) LPFC networks were generated for 6 individuals from the Natural Scenes Dataset (NSD). (B,C) They exhibited smaller frontoparietal networks versus the group average (Group Average: 35.5%, Individual Average = 23.9% ± 2.5%, one-sample t-test: $t(5) = 11.6$, $p < 0.0001$). Data are presented as mean ± SEM. (D-G) They exhibited higher LPFC association network density versus the group average (Group Average: 2.53, Individual Average=3.08 ± 0.02, one-sample t-test: $t(5) = 19.1$, $p < 0.00001$). (H) 5 of 6 subjects exhibited the anterior LANG-CO-DAN motif. (I,J) They exhibited higher than expected association network density at rostral CO (spin test $p = 0.004$).

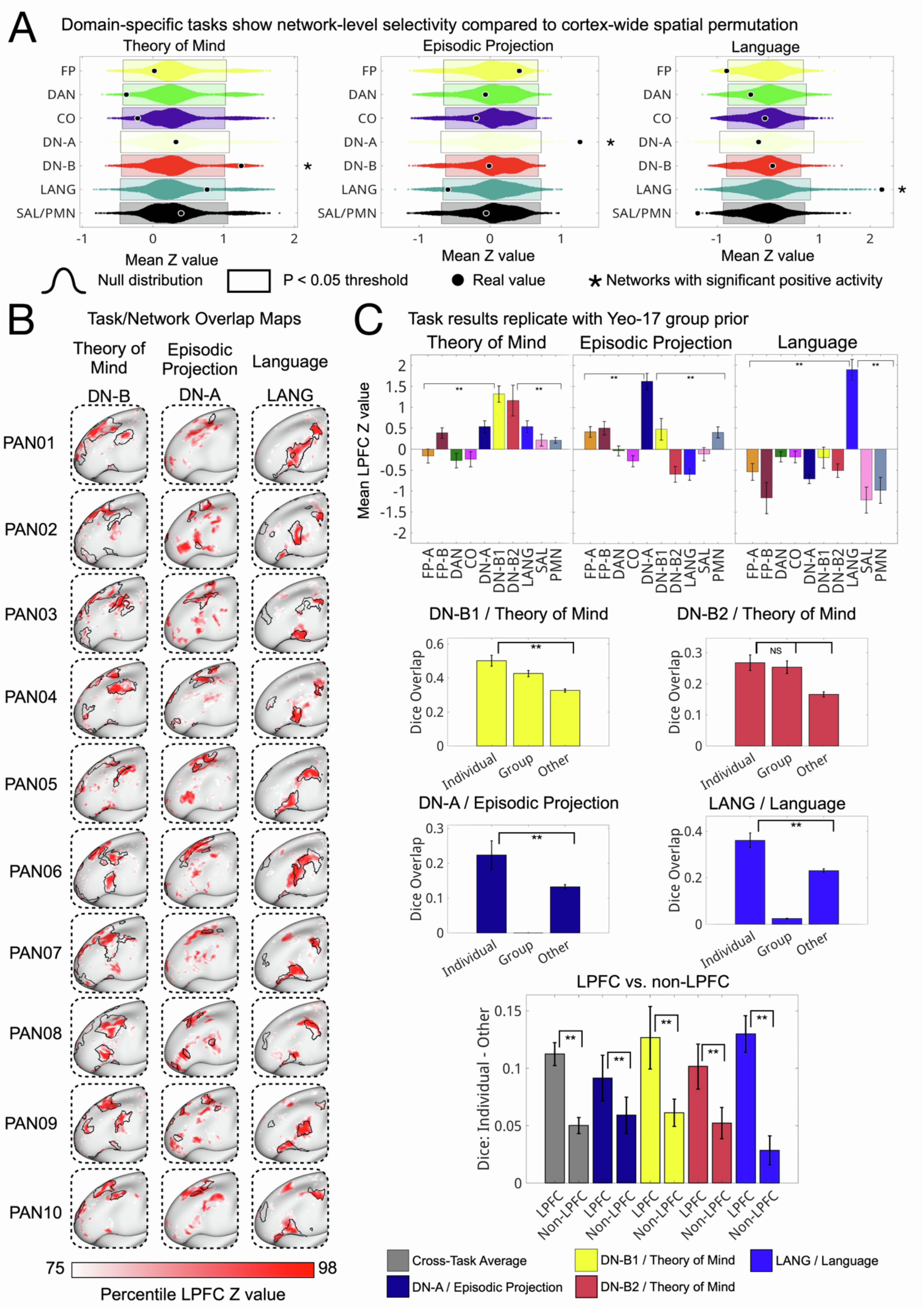
A
Domain-specific tasks show network-level selectivity compared to cortex-wide spatial permutation
Theory of Mind
Episodic Projection
Language
FP
DAN
CO
DN-A
DN-B
LANG
SAL/PMN
Mean Z value
Null distribution
P < 0.05 threshold
Real value
* Networks with significant positive activity
B
Task/Network Overlap Maps
Theory of Mind
Episodic Projection
Language
DN-B
DN-A
LANG
PAN01
PAN02
PAN03
PAN04
PAN05
PAN06
PAN07
PAN08
PAN09
PAN10
75
98
Percentile LPFC Z value
C
Task results replicate with Yeo-17 group prior
Mean LPFC Z value
FP-A
FP-B
DAN
CO
DN-A
DN-B1
DN-B2
LANG
SAL
PMN
DN-B1 / Theory of Mind
DN-B2 / Theory of Mind
DN-A / Episodic Projection
LANG / Language
Dice Overlap
Individual
Group
Other
NS
LPFC vs. non-LPFC
Dice: Individual - Other
LPFC
Non-LPFC
Cross-Task Average
DN-B1 / Theory of Mind
LANG / Language
DN-A / Episodic Projection
DN-B2 / Theory of Mind

Supplemental Figure S5. Networks show task-selectivity compared to a spatial permutation test. Task results are replicated using the Yeo-17 group prior. Related to Figures 5 and 6.
(A) Unthresholded task activation maps for theory of mind, episodic projection, and language demands were randomly rotated around the surface 10,000 times and their network-level activations calculated to create a null model of network activity based on the shape and size of the networks. For theory of mind, only DN-B showed significant activity, for episodic projection, only DN-A showed significant activity, and for language, only LANG showed significant activity ($p < 0.05$). (B) The top 25% task-active LPFC vertices (ranked by z-statistic) are shown for all individuals for theory of mind, episodic projection, and language tasks, overlaid with individual-specific borders for the relevant network (DN-B, DN-A, LANG). (C) Network-level task preferences were replicated using the Yeo-17 group prior. Theory of mind preferentially activated default B1 and B2 (all comparisons, corrected $p < 0.01$). Episodic projection preferentially activated default A (all comparisons, corrected $p < 0.01$). Language processing preferentially activated the language network (all comparisons, corrected $p < 0.01$). Task activations showed significantly greater overlap with individual-specific LPFC networks than with either group-average networks or non-specific individual networks (corrected $p < 0.05$ for all comparisons). This effect was larger in the LPFC than in non-LPFC cortical regions ($p < 0.01$). Data are presented as mean ± SEM.

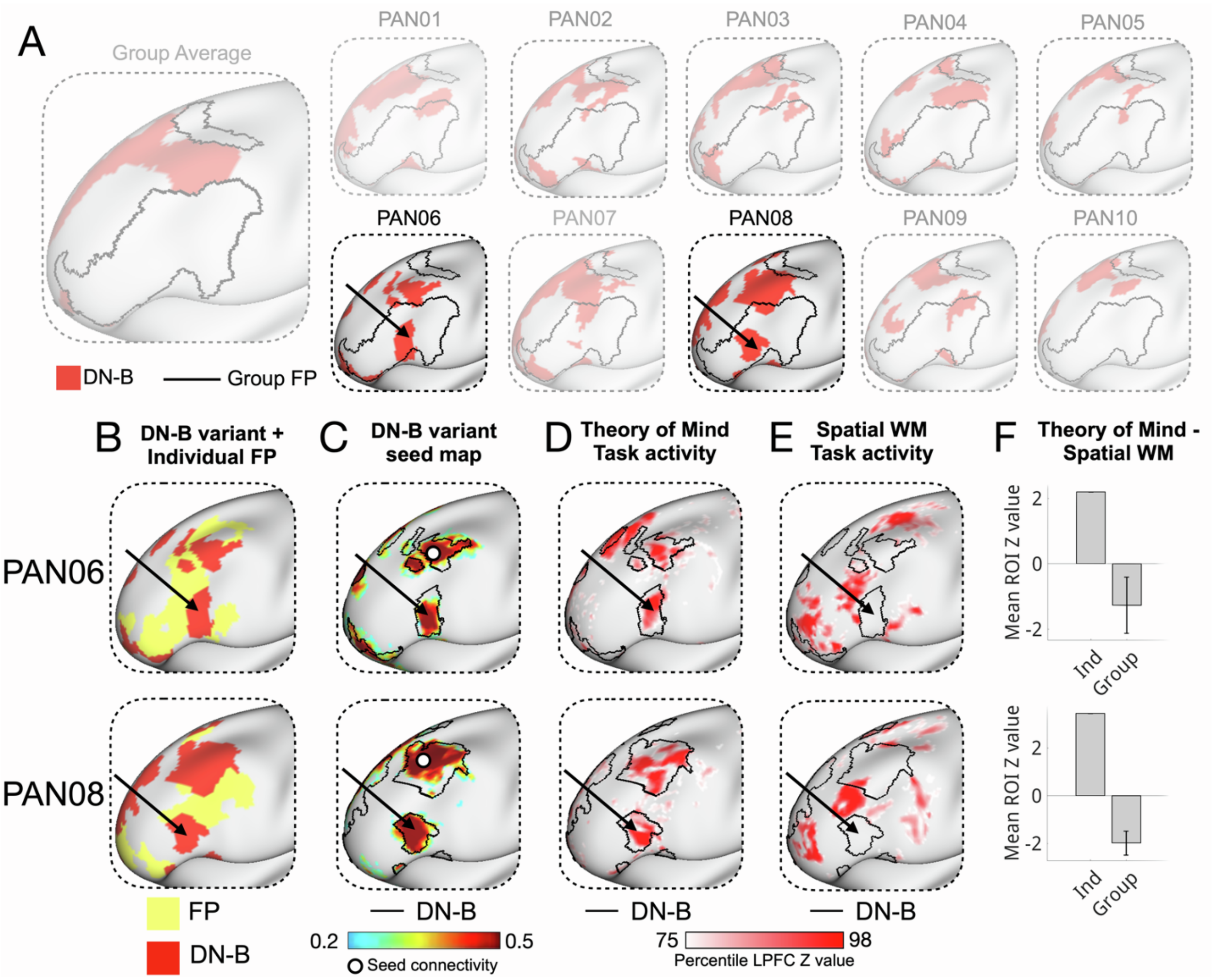
A
Group Average
PAN01
PAN02
PAN03
PAN04
PAN05
PAN06
PAN07
PAN08
PAN09
PAN10
DN-B
Group FP
B
DN-B variant + Individual FP
C
DN-B variant seed map
D
Theory of Mind Task activity
E
Spatial WM Task activity
F
Theory of Mind - Spatial WM
PAN06
PAN08
Mean ROI Z value
2
0
-2
Ind
Group
FP
DN-B
0.2
0.5
Seed connectivity
75
98
Percentile LPFC Z value

Supplemental Figure S6. Idiosyncratic default B regions are embedded within canonical frontoparietal territory in a subset of individuals. Related to Figure 7. (A) Default B network parcellations for 10 individuals and the group average, overlaid with the group-average frontoparietal (FP) network. Two individuals (PAN06, PAN08) showed large default B regions in mid-LPFC, well outside typical default B territory. (B) These variant default B regions were interdigitated with individual-specific FP regions. (C) Seed-based connectivity from the variant regions showed coupling with canonical LPFC default B network regions, supporting their network identity. (D) Theory of mind task activations showed positive responses in the variant default B regions but not in adjacent FP regions. (E) Spatial working memory activations showed the opposite pattern: activation in adjacent FP regions but not in the variant default B regions. (F) Theory of mind > spatial working memory *z*-values are shown for the variant individuals and for the same ROI locations averaged across the eight non-variant individuals. Variant-region ROIs in non-variant individuals did not show theory of mind > spatial WM responses, confirming their individual-specific nature. Data are presented as mean ± SEM.

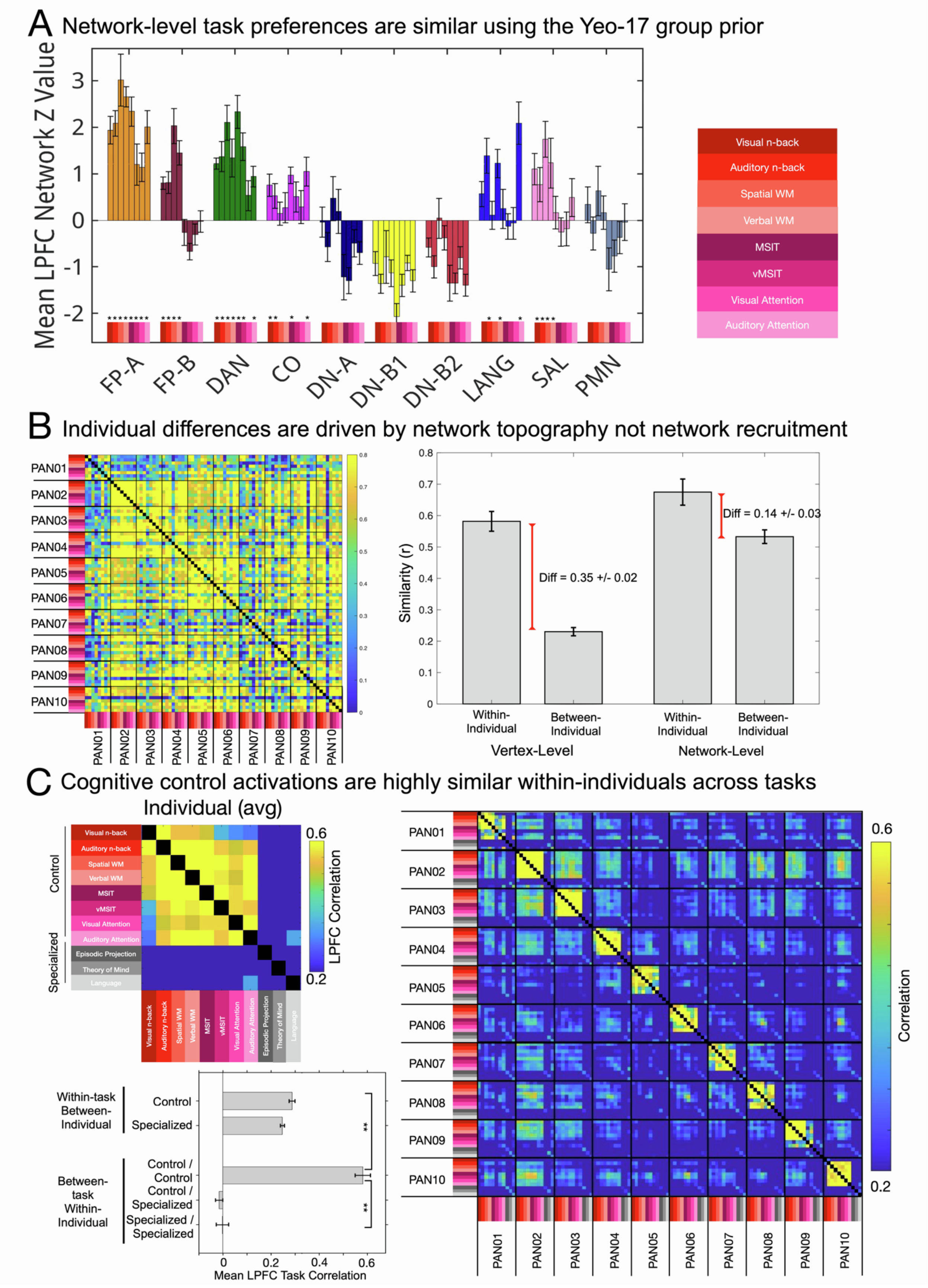

A Network-level task preferences are similar using the Yeo-17 group prior
Mean LPFC Network Z Value
FP-A
FP-B
DAN
CO
DN-A
DN-B1
DN-B2
LANG
SAL
PMN
Visual n-back
Auditory n-back
Spatial WM
Verbal WM
MSIT
vMSIT
Visual Attention
Auditory Attention
B Individual differences are driven by network topography not network recruitment
Similarity (r)
Diff = 0.35 +/- 0.02
Diff = 0.14 +/- 0.03
Within-Individual
Between-Individual
Vertex-Level
Network-Level
C Cognitive control activations are highly similar within-individuals across tasks
Individual (avg)
Control
Specialized
Episodic Projection
Theory of Mind
Language
LPFC Correlation
Within-task Between-Individual
Between-task Within-Individual
Control / Control
Control / Specialized
Specialized / Specialized
Mean LPFC Task Correlation
Correlation

Supplemental Figure S7. Network-level task effects are replicated using the Yeo-17 group prior. Individual task activation differences are driven by differences in network topography rather than network recruitment. Within-individual control activation patterns are highly similar across control tasks. Related to Figure 8.

(A) Network-level task activations were calculated using individualized networks derived from the Yeo-17 group prior. The dorsal attention (DAN), frontoparietal-A (FP-A), frontoparietal-B (FP-B), and cingulo-opercular (CO) networks were the most consistently engaged across tasks (8/8, 8/8, 4/8 and 4/8 tasks, respectively). Language and salience networks were also selectively engaged (3/8 and 4/8 tasks). Significant activations (corrected $p < 0.05$) are marked with asterisks. (B) Average LPFC network activations were calculated for each network for each individual and their patterns were correlated. Vertex-level task activation maps were much more similar across tasks for a single individual than between individuals (between-task: within-subject $r = 0.58 \pm 0.03$, between-subject $r = 0.23 \pm 0.01$, difference = 0.35 ± 0.03). Network-level task activations were also more similar within versus between subjects, but the difference was much smaller than in vertex-wise comparisons (within-subject $r = 0.67 \pm 0.04$, between-subject $r = 0.53 \pm 0.02$, difference = 0.14 ± 0.01, vertex-wise difference > network-wise difference, paired t-test $t(9) = 16.0$, $p < 0.000001$). This suggests that the interindividual differences observed in LPFC activation patterns to the same task are driven more by interindividual differences in network topography than by differences in network recruitment. (C) Cognitive control maps were quite similar to one another ($r = 0.58 \pm 0.03$), more so than they were to domain-specific activation maps within the same individual ($r = -0.02 \pm 0.02$, paired t-test $t(9) = 27.2$, $p < 0.00001$), or than domain-specific maps were to one another within the same individual ($r = 0.00 \pm 0.03$, paired t-test $t(9) = 11.4$, $p < 0.00001$). Across individuals and within-task, both domain-specific activation maps and cognitive control activations showed modest correlations ($r = 0.25 \pm 0.01$, $r = 0.27 \pm 0.03$, respectively). An average correlation matrix is shown across individuals, and all individuals are shown. Data are presented as mean ± SEM.

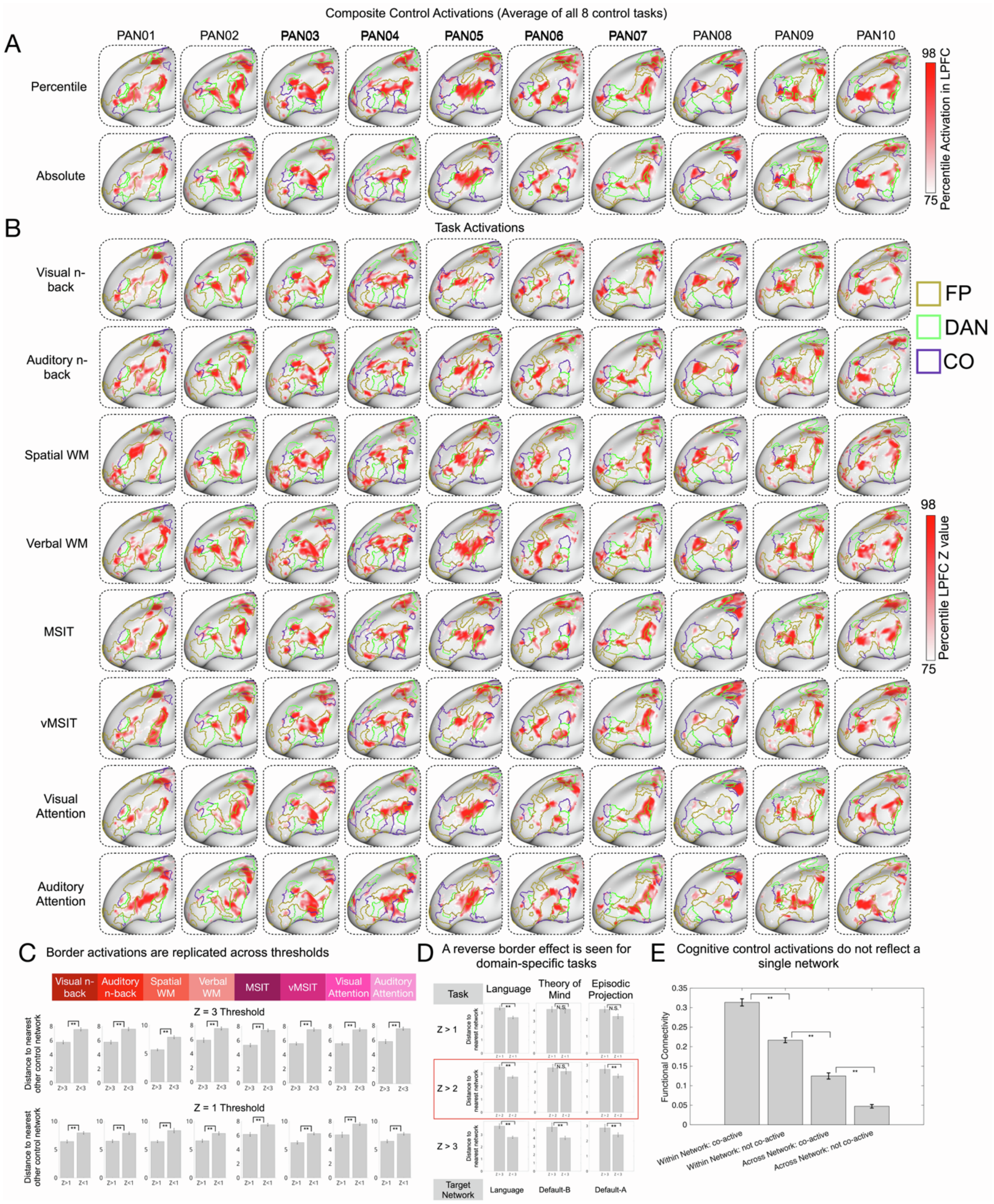

Composite Control Activations (Average of all 8 control tasks)
A
PAN01
PAN02
PAN03
PAN04
PAN05
PAN06
PAN07
PAN08
PAN09
PAN10
Percentile
Absolute
98
75
Percentile Activation in LPFC
B
Task Activations
Visual n-back
Auditory n-back
Spatial WM
Verbal WM
MSIT
vMSIT
Visual Attention
Auditory Attention
FP
DAN
CO
98
75
Percentile LPFC Z value
C
Border activations are replicated across thresholds
Visual n-back
Auditory n-back
Spatial WM
Verbal WM
MSIT
vMSIT
Visual Attention
Auditory Attention
Z = 3 Threshold
Z = 1 Threshold
Distance to nearest other control network
D
A reverse border effect is seen for domain-specific tasks
Task
Language
Theory of Mind
Episodic Projection
Z > 1
Z > 2
Z > 3
Target Network
Language
Default-B
Default-A
E
Cognitive control activations do not reflect a single network
Functional Connectivity
Within Network: co-active
Within Network: not co-active
Across Network: co-active
Across Network: not co-active

Supplemental Figure S8. Cognitive control maps for all individuals. Border activation results replicate across multiple z thresholds and are not present in domain-specific tasks. Cognitive control maps do not reflect a single highly-connected network. Related to Figure 8.

(A) Composite cognitive control maps were created by averaging either the absolute or percentile activation values for each control task for each individual. They show clear differences across people. (B) Cognitive control maps are shown for each task for each individual. The top 25% activated LPFC vertices are shown with the outlines of the FP, CO, and DAN networks. As described in the main text, activation maps revealed a distributed set of highly active regions which were similar across the 8 different tasks, but unique between people. These regions tended to be located near or crossing the borders of the FP, CO, and DAN networks. (C) For each task, LPFC vertices in the FP, CO, and DAN networks were split into "active" ($z > 3$, $z > 2$, $z > 1$) or less-active ($z < 3$, $z < 2$, $z < 1$) groups, and the mean geodesic distance from those vertices to the other two control networks was calculated. Indicated by asterisks, the more active group was significantly closer to the other two control networks (all $p < 0.01$) across all 8 tasks across all 3 thresholds. $Z > 2$ is shown in the main text. (D) This analysis was repeated for domain-specific tasks. The geodesic distance from each LPFC vertex in the target networks (Language: Language, Episodic Projection: Default A, Theory of Mind: Default B) was calculated to the nearest other network. For language and episodic projection demands, more active vertices within the target network were significantly farther from other networks than less active vertices ($p < 0.01$). For theory of mind demands there was no significant relationship at $z > 1$ and $z > 2$, with a significant reversed effect only at $z > 3$. Stars mark $p < 0.01$. (E) For each cognitive control task, vertices within FP, CO, and DAN were classified as active ($z > 2$) or not active ($z < 2$). Resting-state functional connectivity was measured between all vertex pairs, grouped according to (1) shared network membership and/or (2) co-activation status. A graded pattern of functional connectivity emerged: (1) co-active and in the same network > (2) not co-active but in the same network > (3) co-active in different networks > (4) not co-active in different networks. Starred comparisons indicate $p < 0.01$. Data are presented as mean ± SEM.

Theory of Mind Comparisons (Target Network DN-B > Comparison Network)

Comparison Network	Raw p-value	Corrected p-value	T-stats	Cohen's d
FP	3.4e-05	**0.0002**	6.9	2.2
DAN	9.2e-07	**5.5e-06**	10.8	3.4
CO	5.6e-06	**3.35e-05**	8.7	2.8
DN-A	0.00014	**0.0008**	5.7	1.8
LANG	0.0015	**0.009**	4.0	1.3
SAL/PMN	7.2e-06	**4.3e-05**	8.4	2.7

Episodic Projection Comparisons (Target Network DN-A > Comparison Network)

Comparison Network	Raw p-value	Corrected p-value	T-stats	Cohen's d
FP	6.4e-06	**3.8e-05**	8.6	2.7
DAN	5.9e-07	**3.6e-06**	11.4	3.6
CO	1.9e-07	**1.2e-06**	13.0	4.1
DN-B	1.5e-06	**9.1e-06**	10.2	3.2
LANG	1.9e-07	**1.1e-06**	13.1	4.1
SAL/PMN	1.6e-07	**9.4e-07**	13.3	4.2

Language Processing Comparisons (Target Network LANG > Comparison Network)

Comparison	Raw p-value	Corrected p-value	T-stats	Cohen's d
FP	8.1e-06	**4.8e-05**	8.3	2.6
DAN	4.0e-05	**0.00024**	6.8	2.1
CO	0.00012	**0.00070**	5.9	1.9
DN-A	8.2e-06	**4.9e-05**	8.3	2.6
DN-B	4.5e-06	**2.7e-05**	8.9	2.8
SAL/PMN	5.6e-06	**3.4e-05**	8.7	2.8

Supplemental Table S1. Corrected and uncorrected p-values, t-stats and Cohen's d for all network task activation comparisons to high level task domains. Related to Figure 5. Bolded corrected p-values are significant (corrected $p < 0.05$).

Language Network/Language Task Overlap (Individual versus Comparison)

Comparison	Raw p-value	Corrected p-value	T-stats	Cohen’s d
Group	0.0060	**0.012**	3.1	1.0
Others	0.00068	**0.0014**	4.6	1.5

Default Network A /Episodic Projection Task Overlap

Comparison	Raw p-value	Corrected p-value	T-stats	Cohen’s d
Group	0.00087	**0.0017**	4.4	1.5
Others	0.018	**0.036**	2.5	0.8

Default Network B /Theory of Mind Task Overlap

Comparison	Raw p-value	Corrected p-value	T-stats	Cohen’s d
Group	0.00068	**0.0014**	4.6	1.5
Others	1.9e-05	**3.8e-05**	7.5	2.5

LPFC vs. Non-LPFC Network/Task Overlap

Network/Task	Raw p-value	Corrected p-value	T-stats	Cohen’s d
Language/Language	0.00015	**0.00015**	5.7	1.9
DN-A/Episodic Projection	0.027	**0.027**	2.2	0.74
DN-B/Theory of Mind	0.00031	**0.00031**	5.1	1.7
Cross Task Average	2.7e-05	**2.7e-05**	7.2	2.4

Supplemental Table S2. Corrected and uncorrected p-values, t-stats and Cohen’s d for all network task activation overlap comparisons. Related to Figure 6. Bolded corrected p-values are significant (corrected $p < 0.05$).

Task	Network	Mean Z Value	T-stat	Raw p-value	Cohen's d	Corrected p-value
Visual N-back	**FP**	1.41	7.25	0	2.29	**0.0002**
	**DAN**	1.58	10.34	0	3.27	**0**
	**CO**	1.05	6.77	0	2.14	**0.0003**
	DN-A	0.18	0.47	0.33	0.15	1
	DN-B	-0.76	-4.26	1.0	-1.35	1
	LANG	0.47	2.16	0.030	0.68	0.2081
	**SAL/PMN**	1.03	4.98	0.0004	1.57	**0.0027**
Auditory N-back	**FP**	1.55	9.5	0	3	**0**
	**DAN**	1.55	5.05	0.0003	1.6	**0.0024**
	**CO**	0.93	4.15	0.0013	1.31	**0.0088**
	DN-A	-0.42	-1.2	0.87	-0.38	1
	DN-B	-1	-6.46	1.0	-2.04	1
	**LANG**	1.2	3.79	0.0021	1.2	**0.015**
	SAL/PMN	0.34	1.24	0.12	0.39	0.8582
Spatial WM	**FP**	2.81	6.31	0.0001	1.99	**0.0005**
	**DAN**	2.78	6.74	0	2.13	**0.0003**
	CO	0.61	2.36	0.021	0.75	0.1483
	DN-A	0.91	1.56	0.077	0.49	0.5356
	DN-B	-0.28	-0.64	0.73	-0.2	1
	LANG	0.2	0.6	0.28	0.19	1
	**SAL/PMN**	1.64	4.53	0.0007	1.43	**0.005**
Verbal WM	**FP**	2.27	9.11	0	2.88	**0**
	**DAN**	2	5.41	0.0002	1.71	**0.0015**
	CO	0.67	1.96	0.041	0.62	0.28
	DN-A	0.17	0.4	0.35	0.13	1
	DN-B	-0.7	-3.46	1.0	-1.09	1
	**LANG**	1.14	5.24	0.0003	1.66	**0.0019**
	SAL/PMN	1.19	2.4	0.02	0.76	0.14
MSIT	FP	0.31	1.02	0.17	0.32	1
	**DAN**	1.68	4.3	0.001	1.36	**0.007**
	CO	0.55	1.79	0.054	0.57	0.38
	DN-A	-1.23	-4.27	1.0	-1.35	1
	DN-B	-1.39	-6.76	1	-2.14	1
	LANG	-0.24	-1.18	0.86	-0.37	1
	SAL/PMN	-0.24	-0.73	0.76	-0.23	1
vMSIT	**FP**	1.09	4.32	0.001	1.37	**0.0068**
	**DAN**	2.68	13.76	0	4.35	**0**
	**CO**	1.1	7.96	0	2.52	**0.0001**

	DN-A	-1.15	-2.45	0.98	-0.78	1
	DN-B	-1.8	-5.9	1.0	-1.87	1
	LANG	0.15	0.44	0.33	0.14	1
	SAL/PMN	-0.35	-1.05	0.84	-0.33	1
Visual Attention	FP	0.47	1.41	0.095	0.45	0.668
	**DAN**	0.94	3.64	0.0027	1.15	**0.0189**
	CO	0.26	0.94	0.19	0.3	1
	DN-A	-0.65	-2.17	0.97	-0.69	1
	DN-B	-0.79	-4.66	1.0	-1.47	1
	LANG	-0.24	-0.74	0.76	-0.23	1
	SAL/PMN	-0.25	-0.68	0.75	-0.22	1
Auditory Attention	FP	0.98	2.94	0.0083	0.93	0.058
	**DAN**	1.37	6.45	0.0001	2.04	**0.0004**
	**CO**	1.31	5.97	0.0001	1.89	**0.0007**
	DN-A	-0.67	-2.82	1.0	-0.89	1
	DN-B	-1.17	-6.44	1.0	-2.04	1
	**LANG**	1.55	3.57	0.003	1.13	**0.0211**
	SAL/PMN	0.09	0.25	0.41	0.08	1

Supplemental Table S3. Corrected and uncorrected p-values, t-stats and Cohen's d for all LPFC network task activations to individual cognitive control tasks. Related to Figure 8. Bolded corrected p-values are significant (corrected $p < 0.05$).

Supplemental References

1. Lynch, C. J. *et al.* Frontostriatal salience network expansion in individuals in depression. *Nature* **633**, 624–633 (2024).
2. Yeo, B. T. T. *et al.* The organization of the human cerebral cortex estimated by intrinsic functional connectivity. *J Neurophysiol* **106**, 1125–1165 (2011).
